# Advancing automatic speech recognition for low-resource ghanaian languages: Audio datasets for Akan, Ewe, Dagbani, Dagaare, and Ikposo

**DOI:** 10.1016/j.dib.2025.111880

**Published:** 2025-07-11

**Authors:** Isaac Wiafe, Jamal-Deen Abdulai, Akon Obu Ekpezu, Raynard Dodzi Helegah, Elikem Doe Atsakpo, Charles Nutrokpor, Fiifi Baffoe Payin Winful, Kafui Kwashie Solaga

**Affiliations:** Department of Computer Science, University of Ghana, Legon-Accra, Ghana

**Keywords:** Speech-to-text, Speech synthesis, Low-resource languages, Natural language processing, Text-to-speech, Speech datasets

## Abstract

Audio datasets are fundamental to the development of automatic speech-recognition (ASR) systems. However, the availability of a large corpus of audio datasets in low-resource languages (LRLs) is limited. This study addresses this gap by introducing audio speech datasets for five low-resource languages spoken in Ghana and parts of Togo. Specifically, it presents a 5000-hour speech corpus in Akan, Ewe, Dagbani, Dagaare, and Ikposo. Each language corpus includes 1000 h of validated audio speech recorded by their indigenous speakers. These audio recordings are spoken descriptions of 1000 culturally relevant images collected using a custom Android mobile application. To enhance the dataset’s utility in ASR and linguistic research 10 % of the audio recordings for each language were randomly selected and transcribed, resulting in approximately 100 h of transcription per language. This dataset represents a critical resource for preserving and documenting Ghanaian languages. It holds the potential for advancing speech and language technologies in these languages. Creating this audio dataset is the first step towards bridging the technological gap between high- and low-resource languages. Ethical guidelines were strictly followed throughout the data collection process and participants were given incentives for lending their voices to this study.

Specifications Table

**Table:** 

Subject	Natural Language Processing (NLP), Speech-To-Text (STT), Automatic Speech Recognition (ASR), Neural Machine Translation (NMT)
Specific subject area	Automatic Speech Recognition for Low-Resource Languages
Type of data	Audio (all recordings are in MP3 format with a sample rate of 44.1 kHz), text (audio transcriptions), images
Data collection	Audio recordings of image descriptions were collected using an Android mobile application. Indigenous speakers of each language downloaded the app and signed in using credentials provided by the research team. Upon accepting the terms in the consent form, the app allowed them to download the 1000 assigned images and to start recording descriptions of the images in their native language only. Gadgets used: Android mobile phones and tablets, laptops, microphones, and power banks
Data source location	Country: Ghana and Togo Regions: Greater Accra, Central Region, Eastern Region, Volta Region, Northern Ghana, Upper West, Plateaux Region of Togo, Communities: Accra, Tampion, Nanton, Cape Coast, Winneba, Kumasi, Kwahu, Mpraeso, Anloga, Keta, Peki, Akatsi, Ho, Juapong, Kpando, Sogakope, Tamale, Jirapa, Wa, Akposokubi, Akposo Kabo, Atakpame Recording Environments: Outdoor, indoor, offices, cars, buses, studio, unspecified environments.
Data accessibility	The dataset is freely available to the public for research, academic, and educational purposes. The datasets can be assessed at: https://doi.org/10.57760/sciencedb.22298

## 1 Value of the Data

• This dataset provides the first enabling step for the development of ASR technologies for Ghanaian languages. More specifically, the dataset reduces the technological inequality between high-resource and low-resource languages by enabling the development of language technologies such as ASR models for Akan, Ewe, Dagbani, Dagaare, and Ikposo. These models can be adapted for applications such as conversational AIs including question-answering (QA) systems, task-oriented dialogue systems (DS), and chatbots. In addition, since the dataset is a collection of spontaneous speech, it will most likely be more adaptable and versatile for training ASR models for the respective languages.
• The dataset, which also includes 100 h of transcription for each language can be used to develop text-to-speech (TTS) systems that generate culturally relevant and linguistically accurate synthesized speech in the five languages. Additionally, it will provide a valuable resource for researchers working on NLP tasks such as machine translation, sentiment analysis, entity recognition, and text summarization, specifically for low-resource languages. Researchers can leverage this dataset to develop language models that improve language understanding and generation in these languages. Thus, this dataset supports a range of language-specific applications in both TTS and NLP.
• The dataset can be integrated into multilingual AI systems to train models that are capable of handling multiple languages simultaneously or transitioning seamlessly between languages (code-switching) within the same system. Researchers can use this for tasks such as dialect detection and classification, as well as gain an understanding of how different dialects within the same language group vary phonetically and acoustically.
• The audio and text dataset serves as a primary resource for the Ghanaian and Togolese languages. It will help preserve the linguistic diversity of these languages and contribute to the documentation of languages that are often underrepresented in global digital spaces. By making these languages digitally accessible, the dataset will help maintain their cultural relevance and continuity. Overall, this dataset supports linguistic research, language preservation efforts, cultural documentation, and contributes to language sustainability and prevention of language extinction.
• The dataset will support education and literacy efforts, as it may be used to develop indigenous language learning tools, pronunciation guides, and interactive language courses for speakers and non-speakers of the languages.
• The audio dataset consists of recordings performed in both indoor and outdoor environments. Hence, it provides a diverse range of acoustic environments that reflect real-world conditions. This diversity will facilitate the training of adaptive models that are robust in different environmental conditions

## 2 Background

Despite the plethora of automatic speech recognition (ASR) systems, low-resource languages (LRLs) particularly those spoken in Ghana and the plateaux region of Togo face a significant gap in the development of these technologies. Unlike high-resource languages, the lack of large-scale, high-quality audio datasets in these languages hinders advancements in language technologies, resulting in technological disparities between high-resource and low-resource languages. Socio-culturally, most of these LRLs remain underrepresented in global digital spaces, thus jeopardizing their linguistic preservation and cultural continuity.

Previous attempts to digitalize these languages carry inherent biases and lack the diversity and amount of data required to train ASR models to detect and learn language patterns. For instance, Akan, the second-most spoken language in Ghana [1], has an audio dataset focused on financial inclusion [2]. Similarly, the Bible remains the primary resource for Ewe [3].

This study addresses this gap by introducing a 5000-hour audio dataset consisting of recordings in Akan, Ewe, Dagbani, Dagaare, and Ikposo. The audio samples of each language were collected from their indigenous speakers. This dataset will enable the development of ASR models for these languages. It will also contribute to technological inclusivity, linguistic preservation, and educational advancement.

## 3 Data Description

Ghana is a multilingual country with five officially recognized languages: Akan, Ewe, Ga, Dagaare, and Dagbani [1,4]. Other languages such as Ikposo are among the endangered languages and are one of the Ghana-Togo mountain languages. The dataset is a collection of audio descriptions of 1000 different images from indigenous speakers of Akan, Ewe, Dagaare, Dagbani, and Ikposo. The same 1000 images were described for all languages, and each audio file is between 15 s to 30 s (an average size of approximately 350 KB). The uploaded dataset contains a total of 970,148 audio files (5384.28 h) and 93,262 transcribed audio files (518 h). The overall UGSpeechData dataset size is approximately 336 GB.

**Fig. 1:**
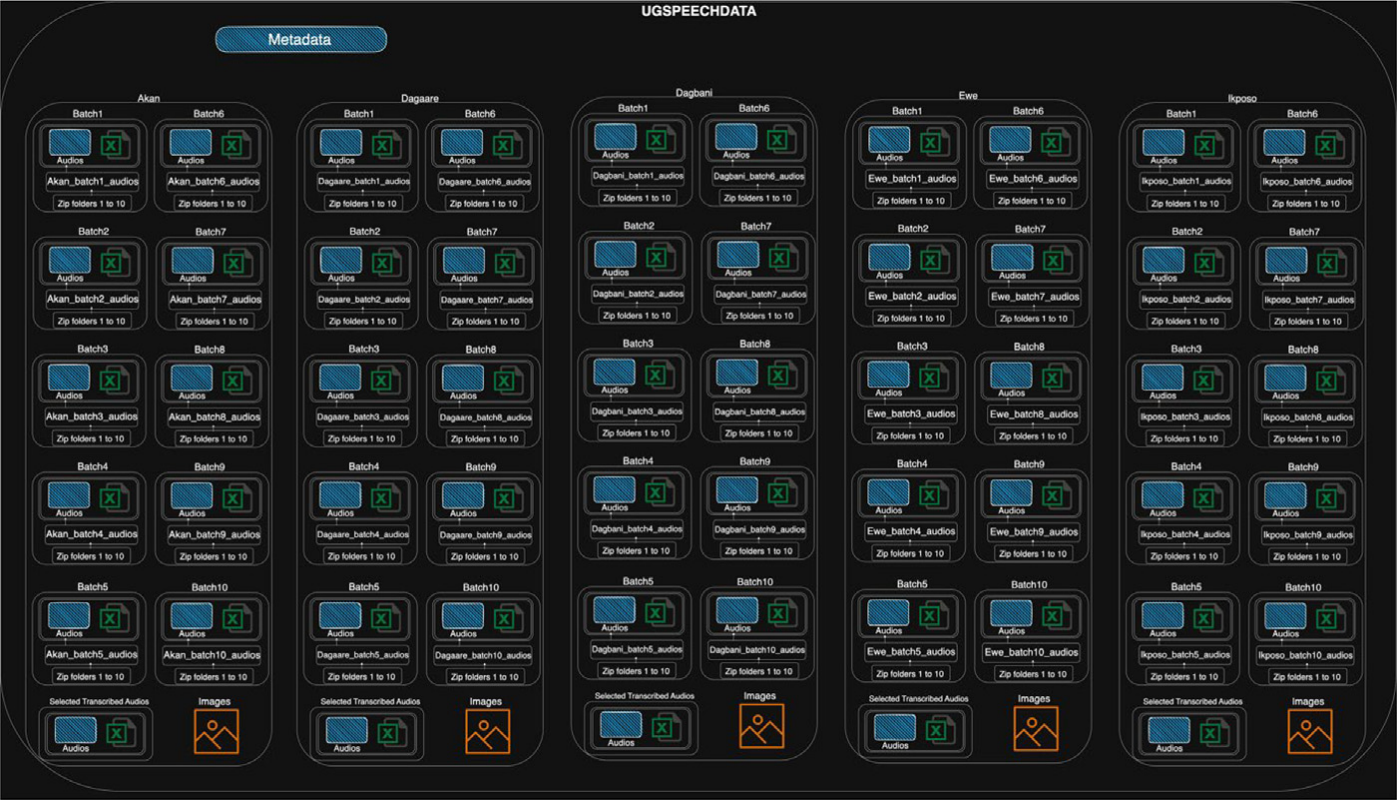
Visual structure of the UGSpeechData repository.

**Fig. 2:**
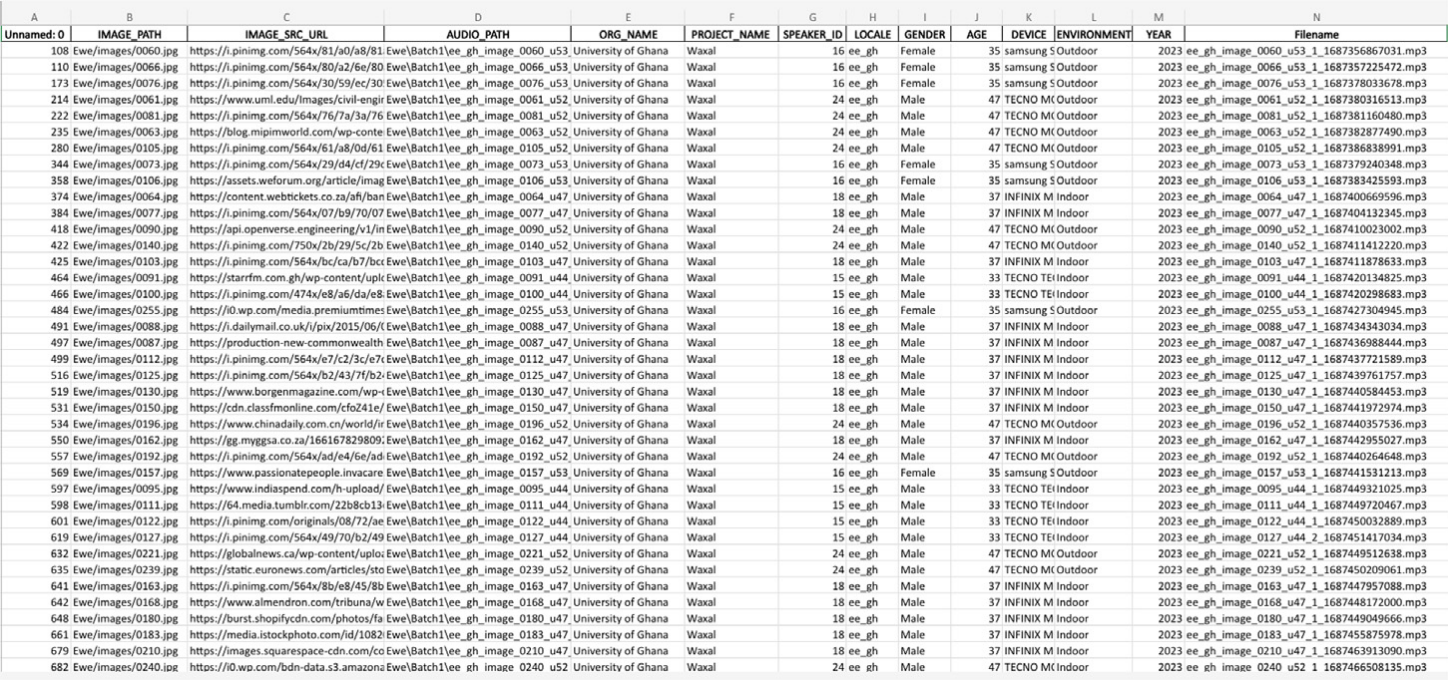
A screenshot of a metadata file.

Fig. 1 presents a visual structure of the UGSpeechData repository. It contains a metadata folder and five language-specific folders namely, Akan, Dagaare, Dagbani, Ewe, and Ikposo respectively. The metadata folder contains five Excel files that are associated with each language, and each file contains 11 sheets corresponding to 11 subfolders (that is, Batch 1 to 10 and selected transcribed audios) of each language folder. Each language folder has 12 subfolders. For example, the Akan folder contains 12 subfolders labeled: *Batch 1 to Batch 10; selected transcribed audios and images*. Each batch contains an *Audios* folder and an *Excel file*. The *Audios* folder contains a subfolder named according to the corresponding batch number (e.g., Akan folder, Batch 1 subfolder contains a subfolder named *Akan_batch1_audios*), which then contains subfolders numbered 1 to 10. More specifically, each *Audios* folder within a *batchX* contains approximately 100 h of audio, and each subfolder within the *Akan+batchX_audios* folder contains 10 h of audio. The Excel file within each *‘batchX’* contains the metadata of the audio clips in that batch (see Fig. 2). The subfolder labeled *‘images’* contains a collection of the 1000 images that study participants described, while the subfolder labeled *'selected audios transcribed'* holds approximately 104.3 h of randomly selected audio recordings which were transcribed and an Excel file containing the corresponding transcription of the audio files and other metadata. This structure is the same for the other four languages. For ease of access and downloads, all subfolders have been zipped.

Refer to Fig. 2 for a screenshot of a metadata file. The metadata provides details of the image path, image source URL, audio path, organization name, project name, speaker ID, locale, speakers’ gender, speakers’ age, device used in recording the audio, the recording environment, the year it was recorded, the full filename, and the shortened filename.

### 3.1 Akan speech corpus

The Akan dataset includes four dialectical variations namely Akuapem Twi, Asante Twi, and Fanti. They were collected from Accra, Kumasi, Kwahu, and Mpraeso. The majority of the recordings were done indoors, followed by outdoors, unspecified environments, offices, and cars. The least number of recordings were done in a studio and none in a bus. The Akan speech corpus is a collection of validated audio descriptions of different images from 2,151 Akan speakers. It includes 195,285 audio files (equivalent to 1085.63 h), out of which 18,787 audio files (104.38 h) were randomly selected and transcribed. The total size of the Akan speech corpus is 60 GB, encompassing all audio recordings, transcriptions, and metadata files. Table 1 presents details of the Akan speech corpus as found in the repository.

**Table 1:** Summary of accepted and transcribed Akan speech corpus.

Gender	Total no audio of files	Equivalent in hours	Total no of audio files collected by recording environment						
			Indoor	Outdoor	Unspecified	Office	Car	Studio	Bus
Male	121,056	672.53	58,447	52,881	4581	3978	1109	60	0
Female	73,952	411.96	39,954	30,040	1579	2159	209	11	0
Others	277	1.14	3	174	100	0	0	0	0
**Totals**	**195,285**	**1085.63**	**98,404**	**83,095**	**6260**	**6137**	**1318**	**71**	**0**

**Details of Transcribed Files**									

Gender	Total no of audio files	Equivalent in hours	Indoor	Outdoor	Unspecified	Office	Car	Studio	Bus

Male	10,920	60.67	4475	5888	466	91	0	0	0
Female	7867	43.71	5147	2445	35	238	2	0	0
**Totals**	**18,787**	**104.38**	**9622**	**8333**	**501**	**329**	**2**	**0**	**0**

### 3.2 Ewe speech corpus

The Ewe speech corpus consists of various dialectical variations of the language namely Anlo, Tongu, Wedome, and Gbi. They were collected from Accra, Anloga, Keta, Peki, Akatsi, Ho, Juapong, Kpando, and Sogakope. The recordings were done in different environments, but the majority were done outdoors, followed by indoors, in unspecified environments, offices, cars, studios, and buses. The Ewe speech corpus is a collection of validated audio descriptions of images from 1,905 Ewe speakers. It includes 203,391 audio files (1130.76 h) and 19,152 files (106.4 h) of transcribed audio. The total size of the Ewe speech corpus is 61.28 GB, encompassing all audio recordings, transcriptions, and metadata files. Table 2 provides details of the Ewe speech corpus as found in the repository.

**Table 2:** Summary of accepted and transcribed Ewe speech corpus.

Gender	Total no of audio files	Equivalent in hours	Total no of audio files collected by recording environment						
			Outdoors	Indoors	Unspecified	Office	Car	Studio	Bus

Male	121,116	673.98	68,300	46,162	3438	1850	931	412	23
Female	81,684	453.8	49,851	27,513	3317	615	17	250	121
Other	591	2.98	45	494	52	0	0	0	0
**Totals**	**203,391**	**1130.76**	**118,196**	**74,169**	**6807**	**2465**	**948**	**662**	**144**

**Details of Transcribed Files**									

Gender	Total no of audio files	Equivalent in hours	Outdoor	Indoor	Unspecified	Office	Car	Studio	Bus

Male	10,870	60.39	4372	5729	398	210	140	21	0
Female	8282	46.01	4929	3107	114	80	0	52	0
**Totals**	**19,152**	**106.40**	**9301**	**8836**	**512**	**290**	**140**	**73**	**0**

### 3.3 Dagbani speech corpus

The audio recordings for Dagbani were collected from Tamale, Tampion, and Nanton in Northern Ghana. The majority of the recordings were done outdoors, followed by indoors, in unspecified environments, offices, cars, buses, and studios. The Dagbani speech corpus is a collection of validated audio descriptions of images from 1,579 indigenous speakers. It includes 188,805 audio files (1047.21 h) and 18,200 files (101.11 h) of transcribed audios. The total size of the Dagbani speech corpus is 60.54 GB, encompassing all audio recordings, transcriptions, and metadata files. Table 3 provides details of the Dagbani speech corpus as found in the repository.

**Table 3:** Summary of accepted and transcribed Dagbani speech corpus.

Gender	Total no of audio files	Equivalent in hours	Total no of audio files collected by recording environment						
			Outdoor	Indoor	Unspecified	Office	Car	Bus	Studio
Male	157,385	874.36	110,664	42,690	3293	574	48	48	68
Female	30,413	168.96	15,124	12,210	1533	691	490	208	157
Other	1007	3.89	637	0	306	64	0	0	0
**Totals**	**188,805**	**1047.21**	**126,425**	**54,900**	**5132**	**1329**	**538**	**256**	**225**

**Details of Transcribed Files**									

Gender	Total no of audio files	Equivalent in hours	Outdoor	Indoor	Unspecified	Office	Car	Bus	Studio

Male	13,026	72.37	6871	5681	197	198	23	9	47
Female	5130	28.5	2328	2432	161	134	18	38	19
Other	44	0.24	0	0	0	44	0	0	0
**Totals**	**18,200**	**101.11**	**9199**	**8113**	**358**	**376**	**41**	**47**	**66**

### 3.4 Dagaare speech corpus

Dagaare audio recordings were collected from Jirapa and Wa, Upper West Region of Ghana. The majority of the recordings were done outdoors, followed by indoors, and unspecified environments. No recordings were done in offices, studios, cars, and buses. The Dagaare speech corpus is a collection of audio descriptions of images from 461 speakers. It includes 190,684 audio files (1059.33 h) and 18,948 files (105.30 h) of transcribed audio. The total size of the Dagaare speech corpus is 64.81 GB, encompassing all audio recordings, transcriptions, and metadata files. Table 4 provides details of the Dagaare speech corpus as found in the repository.

**Table 4:** Summary of accepted and transcribed Dagaare speech corpus.

Gender	Total no of audio files	Equivalent in hours	Total no of audio files collected by recording environment						
			Outdoor	Indoor	Unspecified	Office	Studio	Car	Bus
Male	112,610	625.61	100,864	11,746	0	0	0	0	0
Female	78,053	433.63	63,690	14,341	20	2	0	0	0
Other	21	0.086	0	0	21	0	0	0	0
**Totals**	**190,684**	**1059.33**	**164,554**	**26,087**	**40**	**2**	**0**	**0**	**0**

**Details of Transcribed Files**									

Gender	Total no of audio files	Equivalent in hours	Outdoor	Indoor	Unspecified	Office	Studio	Car	Bus

Male	11,190	62.17	7697	3491	2	0	0	0	0
Female	7758	43.1	6920	838	0	0	0	0	0
Other	6	0.025	0	0	6	0	0	0	0
**Totals**	**18,948**	**105.30**	**14,617**	**4329**	**8**	**0**	**0**	**0**	**0**

### 3.5 Ikposo speech corpus

Audio recordings for Ikposo were collected from Akposokubi and Akposo Kabo in Ghana and Atakpame in Togo. The majority of the recordings were done outdoors, followed by indoors, offices, unspecified environments, and studios. No recordings were done in cars and buses. The Ikposo speech corpus is a collection of audio descriptions of images from 628 indigenous speakers of Ikposo. It includes 191,983 audio files (1061.35 h) and 18,175 files (100.81 h) of transcribed audio. The total size of the Ikposo speech corpus is 60.67 GB, encompassing all audio recordings, transcriptions, and metadata files. Table 5 provides details of the Ikposo speech corpus as found in the repository.

**Table 5:** Summary of accepted and transcribed Ikposo speech corpus.

Gender	Total no of audio files	Equivalent in hours	Total no of audio files collected by recording environment						
			Outdoor	Indoor	Office	Unspecified	Studio	Car	Bus
Male	98,469	547.04	83,809	11,095	1390	1275	900	0	0
Female	89,749	498.62	81,936	6380	1225	208	0	0	0
Other	3765	15.69	346	0	0	3419	0	0	0
**Totals**	**191,983**	**1061.35**	**166,091**	**17,475**	**2615**	**4902**	**900**	**0**	**0**

**Details of Transcribed Files**									

Gender	Total no of audio files	Equivalent in hours	Outdoor	Indoor	Office	Unspecified	Studio	Car	Bus

Male	8712	48.40	7744	735	91	142	0	0	0
Female	9346	51.92	8257	1013	11	65	0	0	0
Other	117	0.49	91	0	0	26	0	0	0
**Totals**	**18,175**	**100.81**	**16,092**	**1748**	**102**	**233**	**0**	**0**	**0**

## 4 Experimental Design, Materials and Methods

In contrast to existing language audio datasets, where professionals read existing transcripts of the language from specific sources [[5], [6], [7]], this study provided participants with images and asked them to describe them in their own words. This approach was adopted to facilitate spontaneous and natural speech, as well as the collection of language’s diverse intonations and variations more authentically. This was achieved using a controlled crowdsourcing solution. Specifically, we developed and used an Android app (UGSpeechData app) to collect audio speech data directly from indigenous speakers of each language in their natural environment.

**Fig. 3:**
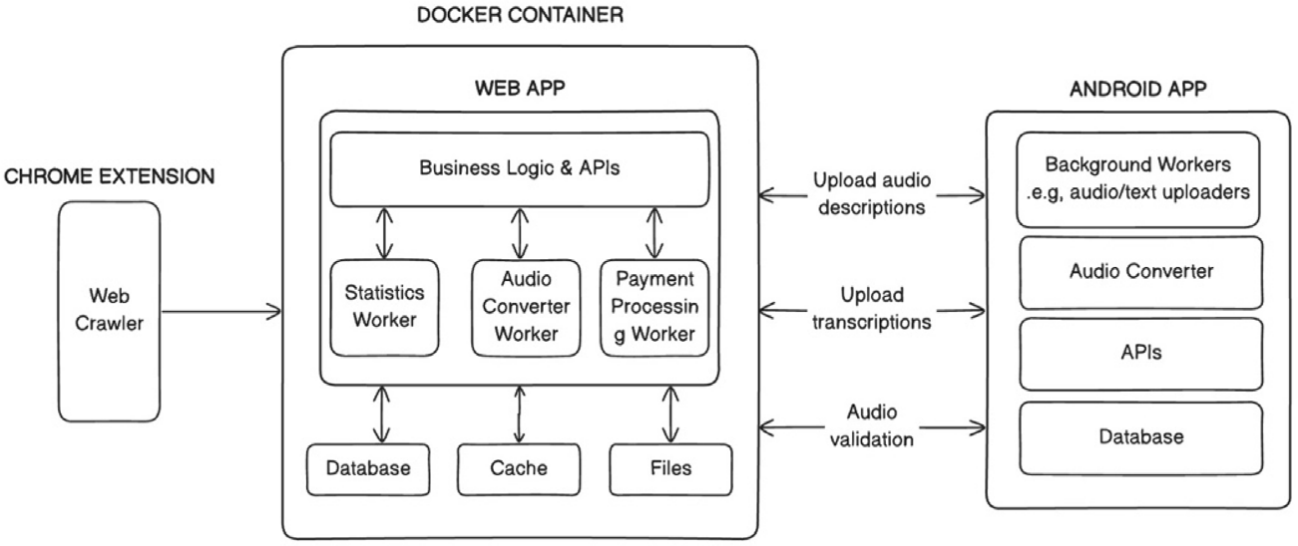
System architecture diagram.

The UGSpeechData app comprises three systems: a web crawler, a web application, and an Android app (see Fig. 3). The web crawler extracted images from websites and populated the image database. The web application and Android app provided interfaces for admins and volunteers to manage, contribute, and validate audio recordings. These interfaces were designed to ensure real-time monitoring of the audio data collection and other related activities as well as minimize potential errors that may arise from human collation.

The data processes are mainly done by the docker container in Fig. 3. The process begins on the user’s device, where audio is recorded in .wav format to allow real-time feedback features such as long-pause detection and ambient noise alerts. Once the recording was completed, the audio was converted into .mp3 using ffmpeg. In over 99.5 % of cases, the conversion was successful and the .mp3 was uploaded to the server. In rare instances (approx. 0.5 %), the Android OS on low-resource devices may terminate the background conversion service. After three failed attempts, the .wav file was uploaded instead.

When the audio got to the server, it was immediately registered in the database along with its metadata. A background worker service then asynchronously processed each uploaded audio file. This service: validated the integrity of uploaded audio files to detect and eliminate corrupted files; converted any remaining .wav files into .mp3; and updated audio records in the database, including replacing .wav file links with the new .mp3 versions and incrementing the count of valid audios per user.

All the data collection processes except participant recruitment were done using the app. They include:

i. Image selection
ii. Screen and select participants
iii. Audio recording
iv. Audio validation
v. Audio transcription
vi. Pay incentives to participants (recorders, validators, and transcribers)

### 4.1 Image selection

To capture a large variety of spoken words in the form of sentences and to overcome the challenges of manually performing sentence segmentation of audio data, participants were required to describe a set of images in their local dialect. Thus, a database of culturally relevant images was curated into the UGSpeechData app. A web crawler was used to extract images from websites such as Pinterest and Google images. This created an initial pool of 8,000 images. Stakeholders were engaged and provided with Samsung Tablets to select and validate the images. Selected images were required to be easily describable in at least three different ways between 15 s and 30 s. In addition, images were screened to ensure they were devoid of any nudity or profanity. Context specificity was another crucial consideration; thus, the majority of the selected images needed to resonate with the linguistic and cultural contexts of Ghanaians.

At the end of the image selection process, 1000 images were selected cutting across different categories. All images were 400px by 400px in size. The 1000 selected images and their associated URLs were populated into the SpeechData app image database. The image categories included but were not limited to the following: Love/Romance, Sanitation, Travel/Tourism, Weather/Climate, History/ Ethnicity, Technology, Automobile, Security, Transportation, Architecture/Construction/Furniture, Fashion/clothing, Food/Drink, Trading/commerce, Hospitality, Lifestyle, Health/Medicine, Agriculture, Economy, Entertainment, Arts/crafts, Science, Justice/Law Enforcement, Mining, Education, Governance, Leisure, Immigration, Home/Housing, Religion, Engineering, Accidents/disaster, Sports, Culture, Family, and Nature.

### 4.2 Participant recruitment, screening, and selection

Participants were recruited using convenience sampling, snowball sampling, and social media advertisements and visits by the research team to several villages within Ghana and Togo. Recruitment was done in three batches. We started with Akan and Ewe, then Dagbani and Dagaare, and finally Ikposo speakers. Three main groups of participants were recruited for this study, the recorders (indigenous speakers of each language who volunteered to record descriptions of assigned images), validators (indigenous and proficient speakers of each language who were selected to validate the audios based on predefined rules), and transcribers (language-specific linguist/experts who were selected from language institutions to transcribe samples of the validated audios). All participants were trained on how to use the app for their designated roles. They were provided with predefined guidelines specific to their tasks. Participants downloaded the app and were given unique login credentials (username and password).

**Fig. 4:**
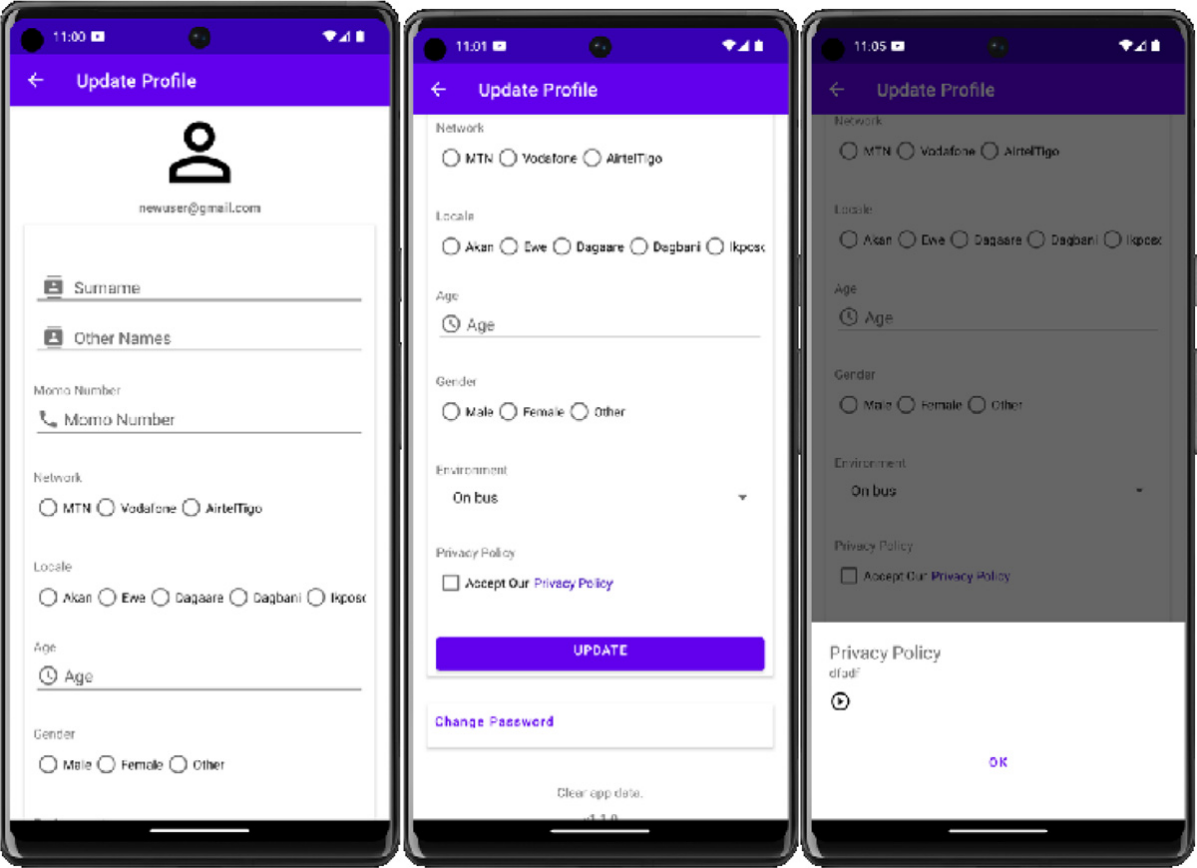
Screenshot of user profile page.

Before recording, recorders were required to sign the consent form and provide relevant demographics, including their indigenous spoken language, age, gender, and recording environment (see Fig. 4). Demographic-related data was used for administrative purposes only and could only be accessed by the principal investigator (the lead author). In addition, the UGSpeechData app automatically retrieved the recording device's name and the timestamp of each recording.

To verify the fluency of recruited recorders in their respective indigenous languages and to ensure that they understood the recording guidelines, a screening process was conducted by selected language experts. Recorders were required to describe their first 10 assigned images using the app, and the language experts reviewed these recordings to evaluate their fluency and adherence to the following guidelines:

i. Recordings must be done in a quiet environment without conflicting sounds.
ii. Recordings must be in the selected language. English words were permitted if no equivalent terms existed in the language. In such instances, local pronunciation of the English word was acceptable.
iii. Recordings must be within 15 s and 30 s without pauses longer than 3 s.
iv. Excessive use of speech mannerisms (e.g., filler words) should be avoided.
v. Descriptions must not start with phrases such as ‘I see, ‘ ‘in this picture,’ or ‘It looks like.’
vi. Descriptions must not include profane or offensive words.

Recorders were selected if their descriptions of at least 8 out of the 10 images adhered to these guidelines. Once selected, restrictions in the app were removed, allowing them to download and record assigned images.

### 4.3 Audio recording

**Fig. 5:**
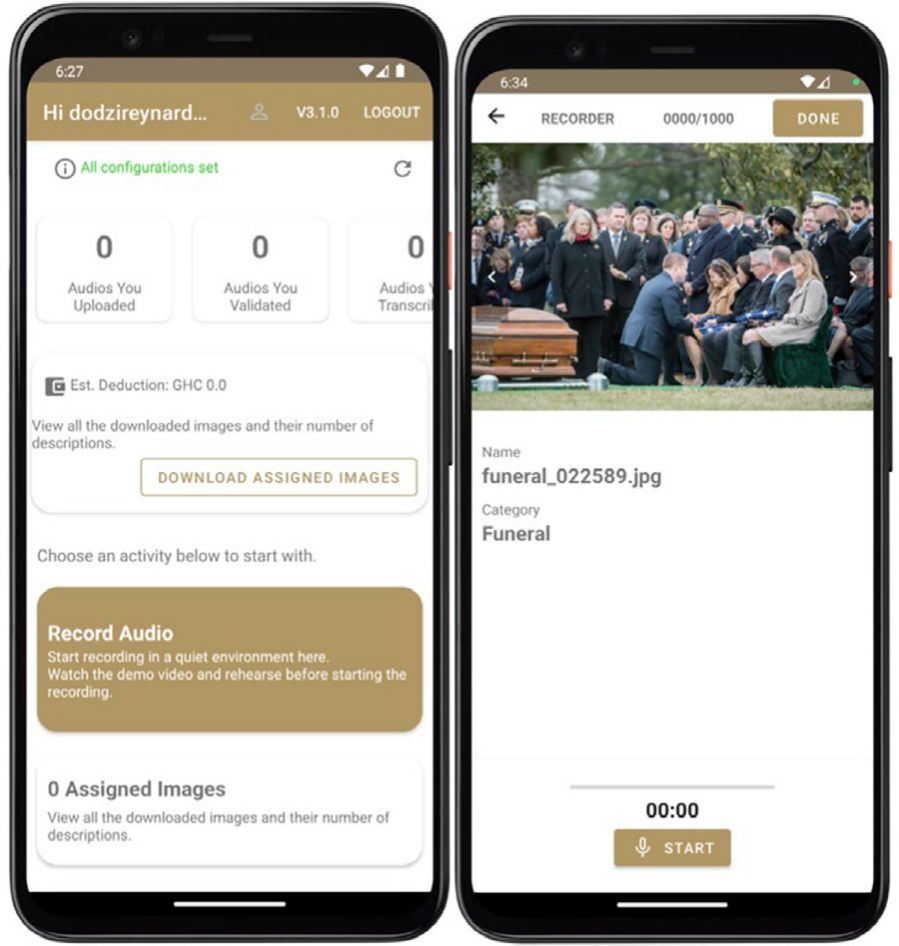
Home and recording page.

Selected recorders were expected to provide audio descriptions of 1000 images using the UGSpeechData mobile app. Upon logging into the app with the provided credentials, recorders downloaded the 1000 assigned images. Before each recording, the app monitored background noise levels. The record button was only activated if no background noise was detected by the app. The app allowed recorders to ‘play,’ ‘save,’ or ‘delete’ their audio descriptions. However, they could only save audio descriptions if it was between 15 s and 30 s; if there were less than three (3) seconds of pause during the description; and if there were no excessive speech mannerisms. The app also prevented duplicate recordings of the same image by a recorder. Fig. 5 shows a screenshot of the UGSpeechData home and recording page.

Upon completion of each recording, the audio files which were initially captured in .wav format were immediately converted to .mp3 format using ffmpeg and uploaded to the UG server. Real-time audio processing, such as the detection of long pauses and ambient noise, necessitated the initial use of .wav for recordings. Converting audio files to .mp3 reduced the average file size from approximately 4 MB to about 350 KB. This enabled faster uploads even in regions with limited network coverage, such as areas with only 3 G connectivity.

Participants who used their devices typically had approximately 500 MB of free storage available, which was sufficient for storing over 1000 audio files. Those who used Tablets provided by the researchers had 32 GB of storage. These limits were never reached per user. During recording, audios were automatically uploaded to the server if the recording device was connected to WIFI or had mobile data turned on. When internet connectivity was unavailable, the audio files were temporarily stored on the device. Once the internet connectivity was restored and the battery of the recording device (tablets or phones) was at least 60 % charged, a background service integrated into the Android app, triggered automatically by the Android OS ensured prompt uploads once connectivity resumed. It is important to note that users had unique IDs, and a Tablet was used to login a new user only when audios from the previous user were uploaded. Once the file was confirmed uploaded and validated by the server, it was deleted from the device storage to maintain sufficient space for continued data collection. This immediate transmission approach ensured minimal local storage consumption.

To maintain security and data integrity, all files (audio and images) were stored in the device's private internal storage. This design choice ensured:

i. Files were inaccessible to other apps, enhancing data privacy.
ii. Users could not access files outside the UGSpeechData app interface.
iii. After successful upload, users had the option to delete files directly within the app; otherwise, deletion could only occur upon uninstalling the app.

Recordings were done in a comfortable environment of the recorders' choosing, with the flexibility to take breaks as needed. Completing descriptions for all 1000 images took approximately four to five hours per recorder. Once a recorder completed their assigned images, they were disabled from further recording. Although 1000 images were assigned to each recorder, completing all descriptions was not mandatory. Recorders had the option to stop at any time. After 8 months of audio data collection, we received approximately 1000,000 audio recordings (5550 h) for all languages. Audios were recorded in different environments namely outdoors, indoors, car, bus, office, studio, and unspecified environments. The next step was to validate the collected audio recordings.

### 4.4 Audio validation

To ensure the reliability and integrity of the collected audio recordings as well as confirm adherence to the predefined guidelines, it was imperative to validate all recordings. Thus, we selected language experts from each language, and they were assigned the role of ‘validators’ in the app. These experts possessed the necessary linguistic expertise and cultural familiarity to accurately assess and determine whether an audio recording should be accepted or rejected. Each validator was assigned 240 audio files at a time and at least two validators reviewed a specific recording. In instances where there was a conflict, that is, a specific audio file was accepted by one validator but rejected by another, such a file was flagged for conflict resolution. Here, a third validator would make the final decision to either accept or reject the audio recording.

**Fig. 6:**
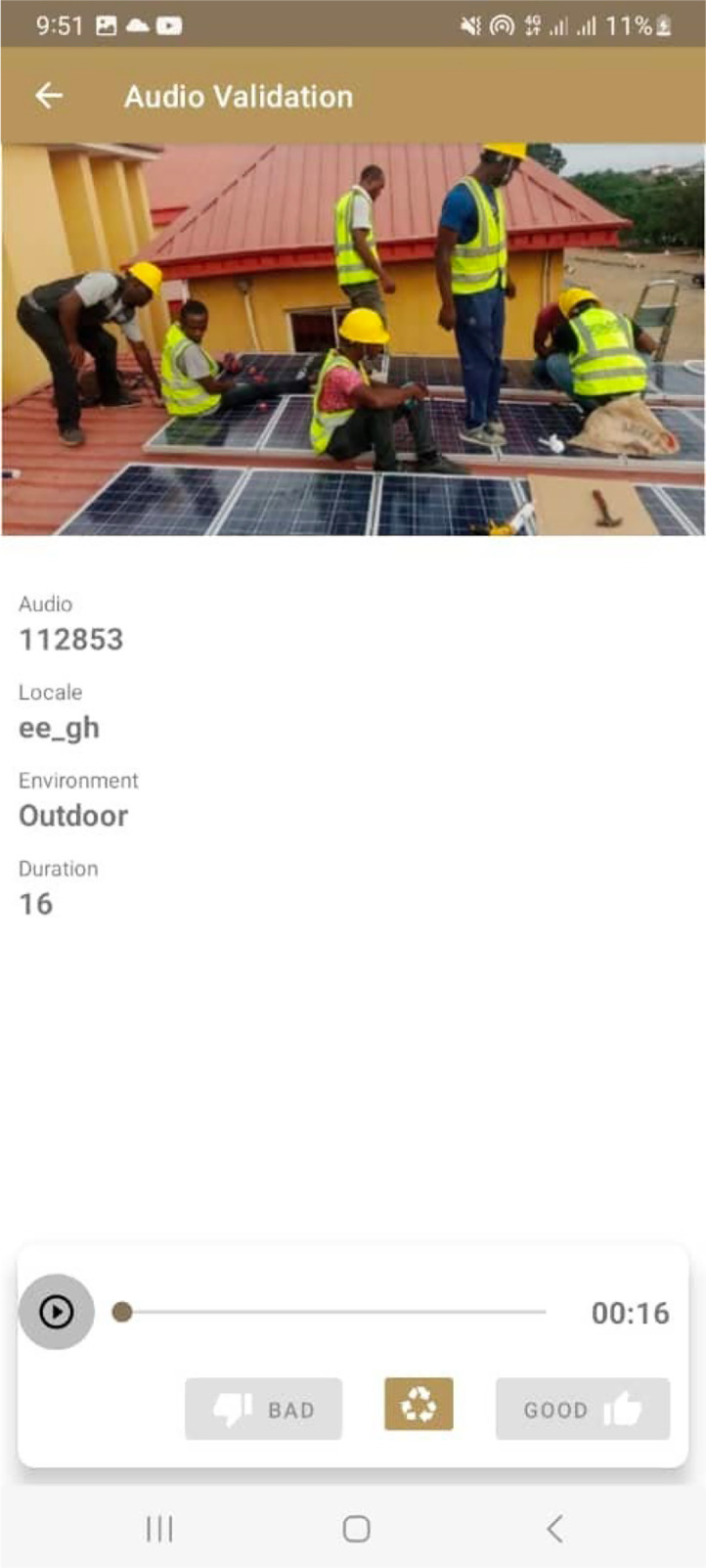
Screenshot of audio validation page.

To retrieve audio files for validation, the validator will select retrieve audios for validation. The app scheduled a background service which downloaded the specified batch of audios to the validator’s device. If the device was already online, downloads commenced immediately. During validation, the audio files were played directly from the local device storage. This enabled smooth, uninterrupted playback regardless of current internet connectivity. Upon submission of the validator’s verdict to the server, each validated audio was automatically and asynchronously deleted from the local storage, freeing device space efficiently. Specifically, they would select ‘good’ if an audio met the predefined guidelines or ‘bad’ if otherwise. Refer to Fig. 6 for a screenshot of the validation page. This approach allowed validators the flexibility to download audio files during periods of stable internet connectivity and subsequently validate them at their convenience, irrespective of ongoing internet access. For example, validators could download audio batches at their workplace and perform validations offline later at home.

At the end of the validation process, approximately 96.92 % of the audio recordings were accepted. Specifically, 970,148 audio files (5384.28 h) from 6,724 unique recorders were accepted (good), while 29,852 audio files (165.72 h) were rejected (bad). Table 6 provides demographic details of accepted audio files/recordings per language.

**Table 6:** Distribution of demographics by gender and age per language.

Languages	Gender	No. of recorders	No. of audio files	Age range	No. of recorders	No. of audio files
Akan	Male	1279	121,056	18–25	1389	92,029
	Female	858	73,952	26–35	466	73,439
	Other	14	277	36–45	164	18,524
**Total**		**2151**	**195,285**	46–55	76	5625
				56–65	33	3816
				66–75	10	1769
				Unspecified	13	83
Ewe	Gender	No. of recorders	No. of audio files	Age range	No. of recorders	No. of audio files
	Male	1076	121,116	18–25	751	56,361
	Female	816	81,684	26–35	606	84,544
	Other	13	591	36–45	287	32,676
**Total**		**1905**	**203,391**	46–55	149	17,175
				56–65	71	9796
				66–75	30	2613
				Unspecified	11	226
Dagbani	Gender	No. of recorders	No. of audio files	Age range	No. of recorders	No. of audio files
	Male	1172	157,385	18–25	849	95,598
	Female	398	30,413	26–35	484	60,876
	Other	9	1007	36–45	189	25,581
**Totals**		**1579**	**188,805**	46–55	43	5785
				56–65	6	555
				66–75	2	104
				Unspecified	6	306
Dagaare	Gender	No. of recorders	No. of audio files	Age range	No. of recorders	No. of audio files
	Male	213	78,053	18–25	144	56,622
	Female	245	112,610	26–35	206	88,880
	Other	3	21	36–45	78	34,677
**Totals**		**461**	**190,684**	46–55	24	8912
				56–65	4	368
				66–75	7	1304
				Unspecified	3	21
Ikposo	Gender	No. of recorders	No. of audio files	Age	No. of recorders	No. of audio files
	Male	329	98,469	18–25	193	55,257
	Female	283	89,749	26–35	119	37,737
	Other	16	3765	36–45	116	42,474
**Totals**		**628**	**191,983**	46–55	106	26,769
				56–65	53	18,500
				66–75	24	7332
				Unspecified	11	3914
**Overall total**		**6724**	**970,148**		**6724**	**970,148**

### 4.5 Audio transcription

Training datasets for automatic speech recognition (ASR) systems require transcribed audio recordings. Hence, 10 % of the validated audio recordings from each language were randomly selected for transcription. Language-specific linguists and prolific writers (hereinafter referred to as transcribers) were selected to transcribe these recordings in the five collected languages. All transcribers were university students and lecturers who were studying or teaching the respective languages at undergraduate or postgraduate levels.

Each language had 10 to 20 transcribers who were assigned audio recordings in their respective indigenous languages. If for technical issues, an audio file in a different language was mistakenly assigned to a transcriber, they were instructed to type "N/A" and skip the file. To ensure consistent and accurate transcriptions across languages, transcribers were trained and provided with a set of strict transcription guidelines. These guidelines were as follows:

i. Use earphones and work in a quiet environment while transcribing.
ii. Listen to 3–4 words at a time, type them, and review the transcription for accuracy. That is, ensure that what you typed matches what you heard.
iii. Do not transcribe audio with significant background noise or secondary voices.
iv. Do not modify or add words to the transcription.
v. Transcribe corrections made by the speaker. For example, if a speaker says, "A red car—oh no, a yellow car", transcribe all spoken words exactly as heard.
vi. Transcribe all comments made by the speaker, regardless of their relevance to the main content.
vii. Transliterate English words into their phonetic equivalents in the local language.
viii. Represent inaudible words with “…”.
ix. Mark audio in other languages or extremely low-quality recordings as "N/A".
x. Consolidate filler sounds (e.g., "errrrr" → "errr"; "hmmmm" → "hmmm").
xi. Retain well-known English words or phrases (e.g., "Worldwide Web") if appropriate.

In addition, to cater to the unique characters of the languages and to enable efficient transcription, transcribers were trained on how to download and use the custom keyboards. For Akan, Dagaare, and Dagbani, the Ghana keyboard (GBoard) app was installed on the laptops of the transcribers and set as their default keyboard. Transcribers select and add their respective languages before they start transcribing.

The GBoard does not include the Ewe and Ikposo alphabets. Thus, we developed specific keyboards for these languages. The keyboard utilizes the standard QWERTY keyboard layout and incorporates all the special characters that are specific to the languages. We also designed an on-screen keyboard for transcribers who prefer a visual representation of the layout or who use touchscreen devices.

**Fig. 7:**
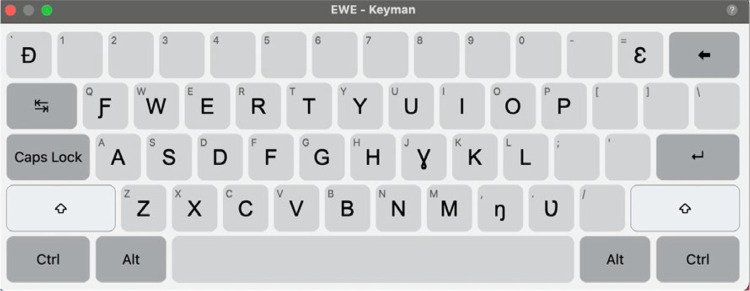
Ewe keyboard.

For Ewe, we incorporated the standard QWERTY keyboard layout optimized for Ewe orthography, including support for all the diacritics and tonal marks. Ewe orthography relies on the African Reference Alphabet and uses diacritics to represent tones (High, Mid, Low) and nasal vowels. The developed Ewe keyboard integrates the following diacritics to represent these tones in writing: the acute accent (‘), the Grave accent (`), the Caron (ˇ), and the Circumflex (ˆ). The Ewe alphabet consists of 30 characters including the 26 letters of the English alphabet, excluding 'c,’ ' j,’ and 'q,’ which are replaced by 'ɔ,’ 'ɣ,’ and 'ƒ,’ respectively. In addition to the standard alphabet, the Ewe keyboard includes these special characters: ɖ, ŋ, ɛ, ɔ, ɣ, ʋ. The keyboard also carters for nasal vowels, indicated by a tilde (˜). Fig. 7 is a visual representation of the Ewe keyboard.

**Fig. 8:**
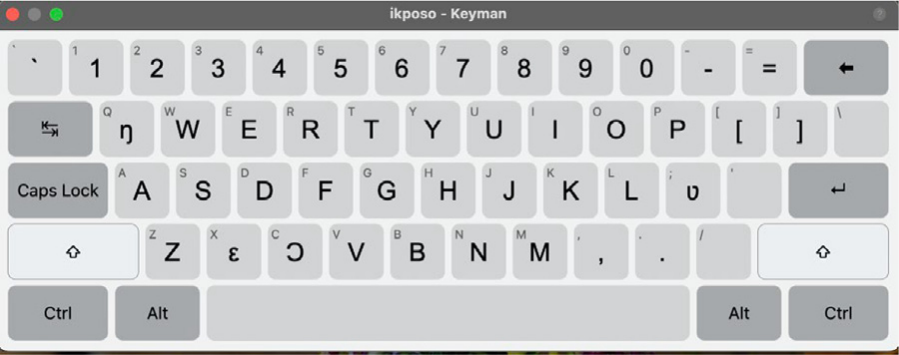
Ikposo keyboard.

Similarly, the Ikposo keyboard uses the QWERTY layout as its foundation. The Ikposo alphabet consists of 27 characters, based on the English alphabet but excluding the letters 'c', 'j', 'q', and 'x' which were replaced by ‘ɔ’, ‘ʅ ’, ‘ŋ ’, and ‘ε’ respectively. The keyboard includes the following special characters essential for writing in Ikposo: ŋ, ʋ, ε, ɔ, and ʅ. Unlike Ewe, Ikposo does not use tonal marks; therefore, the keyboard does not include any diacritics for tones. Fig. 8 is a visual representation of the Ikposo keyboard. Refer to LINK for more details on how to download and use the Ewe and Ikposo keyboard.

**Fig. 9:**
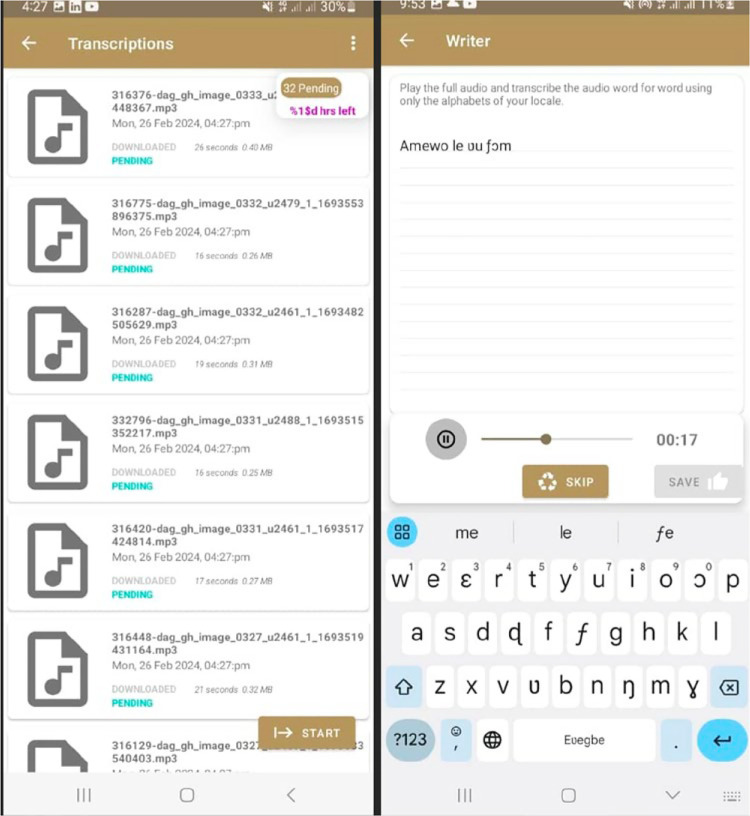
Screenshot of transcription page (Dagaare).

Following the training, transcribers were given login details and assigned the role of ‘Transcribers’ in the app. To manage the workload, the app was configured to assign 240 audio files to each transcriber at a time. Transcribers were required to download and complete these transcriptions within 48 h, after which the app would automatically clear the files and allow the next batch to be downloaded. This process ensured timely completion and equitable distribution of tasks. Fig. 9 is a screenshot of the transcription page.

A total of 93,233 (517.97 h) audio files cutting across the various recording environments and five languages were transcribed. That is, approximately 100 h of the accepted audio files per language were transcribed.

## Limitations

After being trained by the research team, participants were required to know how to use an Android phone and the mobile application independently. However, given the short-term training, not all participants were competent. This may have led to early dropouts for some participants. The UGSpeechData app was only supported by Android. Thus iOS users could not participate in the study. This limitation reduced the inclusivity of the data collection process.

Furthermore, given that the languages have different dialectal variations, the absence of dialectal segmentation during the data collection process poses a limitation in categorizing data based on dialectal differences within the various languages. The dataset by language may be skewed to dialects that had more participants. This limitation may hinder the ability to analyze and interpret data concerning specific dialects. Without the ability to distinguish between dialectal variations, researchers may encounter challenges in accurately representing the linguistic diversity within the languages. Additionally, the lack of dialectal segmentation limits the applicability and relevance of the dataset for studies that specifically focus on dialectal differences and variation.

## Ethics Statement

Ethical approval for this study (which was called WAXAL Project) was obtained from the Ethics Committee for Basic and Applied Sciences (ECBAS), University of Ghana. The participants were duly informed of the objectives of audio data collection and the advantages of having such a dataset. Informed consent was obtained from all the participants (recorders, validators, transcribers, and language experts). Although personal information, such as names and phone numbers, was collected, this information was used for administrative and compensation purposes only. All the participants including enumerators and language leads were duly compensated based on the number of images recorded, audios validated, and audios transcribed. This was done electronically using the UGSpeechData app to their respective mobile money accounts.

## Credit Author Statement

**Isaac Wiafe (Principal investigator):** Conceptualization, Methodology, Data collection, Writing original draft and revisions, Project administration, Supervision, Investigation, Resources, Funding acquisition. **Jamal-Deen Abdulai (Co-PI):** Conceptualization, Methodology, Data collection, Supervision, Investigation. **Akon Obu Ekpezu:** Conceptualization, Methodology, Data collection, Project administration, Investigation, Writing – original draft and revision. **Raynard Dodzi Helegah:** Development and management of data collection tool, Writing original draft, Data collection and management, Investigation. **Elikem Doe Atsakpo:** Management of data collection tool, Data collection and management, Investigation, Writing of original draft. **Charles Nutrokpor:** Management of data collection tool, Data collection and management, Investigation, Writing of original draft. **Fiifi Baffoe Payin Winful:** Data collection and management, Writing original draft. **Kafui Kwashie Solaga:** Data management, development of keyboards, Writing original draft.

## Data Availability

Science Data BankUGSpeechData: A Multilingual Speech Dataset of Ghanaian Languages (Original data).
